# Association between whole grain intake and breast cancer risk: a systematic review and meta-analysis of observational studies

**DOI:** 10.1186/s12937-018-0394-2

**Published:** 2018-09-21

**Authors:** Yunjun Xiao, Yuebin Ke, Shuang Wu, Suli Huang, Siguo Li, Ziquan Lv, Eng-kiong Yeoh, Xiangqian Lao, Samuel Wong, Jean Hee Kim, Graham A. Colditz, Rulla M. Tamimi, Xuefen Su

**Affiliations:** 1 Shenzhen Key Laboratory of Molecular Epidemiology, Shenzhen Center for Disease Control and Prevention, Shenzhen, China; 2 School of Public Health and Primary Care, Faculty of Medicine, School of Public Health, Prince of Wales Hospital, The Chinese University of Hong Kong, Room 508, Shatin, New Territories Hong Kong, China; 3 Alvin J. Siteman Cancer Center and Department of Surgery, Washington University School of Medicine, St. Louis, USA; 4 Channing Division of Network Medicine, Brigham and Women’s Hospital, Harvard Medical School, 181 Longwood Avenue, Boston, MA 02115 USA; 5 Department of Epidemiology, Harvard T.H. Chan School of Public Health, Boston, USA

**Keywords:** Whole grain, Breast cancer, Observational studies, Meta-analysis

## Abstract

**Background:**

Epidemiological studies have found that high whole grain intake may be associated with a reduced risk of breast cancer. However, the evidence has not been consistent. We conducted a meta-analysis to quantitatively assess the association between whole grain intake and breast cancer risk.

**Methods:**

Relevant observational studies were identified by searching PubMed, Embase, Cochrane library databases, and Google Scholar through April 2017. Summary relative risk (RR) estimates were calculated using random-effects meta-analysis.

**Results:**

A total of 11 studies, including 4 cohort and 7 case-control studies and involving 131,151 participants and 11,589 breast cancer cases, were included in the current meta-analysis. The pooled RR of breast cancer for those with high versus low whole grain intake was 0.84 (95% confidence interval [CI]: 0.74 to 0.96, *p* = 0.009; *I*^2^ = 63.8%, *p*_for heterogeneity_ = 0.002). Subgroup analysis by study design found a significant inverse association in the case-control studies (RR: 0.69; 95% CI: 0.56 to 0.87, *p* = 0.001; *I*^2^ = 58.2%, p _for heterogeneity_ = 0.026), but not in the cohort studies (RR, 0.96; 95% CI: 0.82 to 1.14, *p* = 0.69; *I*^2^ = 66.7%, p for heterogeneity = 0.029). In addition, stratified analysis suggested that sample size could be a potential source of heterogeneity.

**Conclusions:**

Results of the current meta-analysis suggest that high intake of whole grains might be inversely associated with a reduced risk of breast cancer, and the inverse association was only observed in case-control but not cohort studies. More large-scale cohort studies are needed to confirm the inverse association observed.

**Electronic supplementary material:**

The online version of this article (10.1186/s12937-018-0394-2) contains supplementary material, which is available to authorized users.

## Background

Breast cancer is the most commonly diagnosed cancer among women worldwide. The incidence rate has been rising increasing over the past several decades [1]. On aggregate, each year 1.7 million women were diagnosed with breast cancer. Most well-established breast cancer risk factors, however, are not easily modifiable such as family history, age at menarche, age at menopause, and reproductive history. Therefore, diet, as a potentially modifiable factor, has been investigated intensively as a potential means for breast cancer prevention [2].

Grains are one of the major staple foods consumed globally and provide 56% of the energy and 50% of the protein intake [3]. They make up the largest proportion of recommended daily food intake in various dietary guidelines. Because of the important role of grains in most diets around the world, the health effects of grain consumption, and in particular whole grains, have attracted much research interest. Whole grains contain endosperm, germ, and bran, in contrast to refined grains, from which germ and bran was removed during the milling process. A high intake of whole grains has been associated with a reduced risk of type 2 diabetes [4–6], cardiovascular disease [7–9], and mortality [8, 10]. In particular, whole grain is a primary source of dietary fiber, which has been associated with a reduced risk of various types of cancer [11]. Two recent meta-analyses reported an inverse association between dietary fiber and whole grain intake and the risk of colorectal cancer [12, 13]. A previous review of mostly case-control studies also reported that higher intake of whole grains was associated with a lower risk of several individual cancers, mainly of the digestive system [14].

The association between whole grain consumption and breast cancer risk has been investigated in previous epidemiological studies. Some have found a possible inverse association [15–21], whereas others have shown no clear association [22–25]. The inconsistent results may be due to different study designs, various dietary intake assessment methods, the amount of whole grain consumption in different study populations, and a range of confounding factors that were adjusted in previous studies. To our knowledge, no systematic review or meta-analysis has been performed to summarize the evidence from observational studies. We, therefore, conducted a meta-analysis to quantitatively evaluate the association between whole grain intake and breast cancer risk.

## Methods

### Data sources and literature search

We followed the guidelines of the Meta-analysis of Observation Studies in Epidemiology group (MOOSE) [26], and the PRISMA criteria guidelines [27], and filled the PRISMA Checklist (Additional file 1: Table S1). Databases including PubMed, EMBASE, Google Scholar, and Cochrane Library were searched through April 2017 for relevant articles that reported the association between whole grain intake and the risk of breast cancer. To avoid missing any relevant studies, we also searched the bibliographies of retrieved papers and recent reviews in the field. The following medical subject headings (MESH) and keywords were used in the literature search, including “grain” or “grains”, “breast cancer” or “breast carcinoma”. We conducted the literature search with combinations of (“grain” and “breast cancer”), or (“grain” and “breast carcinoma”), or (“grains” and “breast cancer”), or (“grains” and “carcinoma”), or ((“grain” or “grains”) and (“breast cancer” or “breast carcinoma”)). No restrictions were imposed.

### Study selection

Studies were eligible if they met the following inclusion criteria: 1) a case-control or cohort study; 2) assessed the association between whole grain intake and the risk of breast cancer; 3) breast cancer cases were diagnosed and verified by pathological biopsies or other standard methods, with controls being females without breast cancer; 4) reported relative risks (RRs) or odds ratios (ORs) and the corresponding 95% confidence intervals (CIs) for the highest versus the lowest levels of whole grain intake.

Two reviewers independently screened the titles and abstracts of the searched papers and excluded the articles which did not meet the above-described inclusion criteria. For those which were difficult to determine the eligibility based on title and abstract review, the full-texts were obtained and reviewed. All disagreements were resolved by discussion to reach a consensus.

**Fig. 1:**
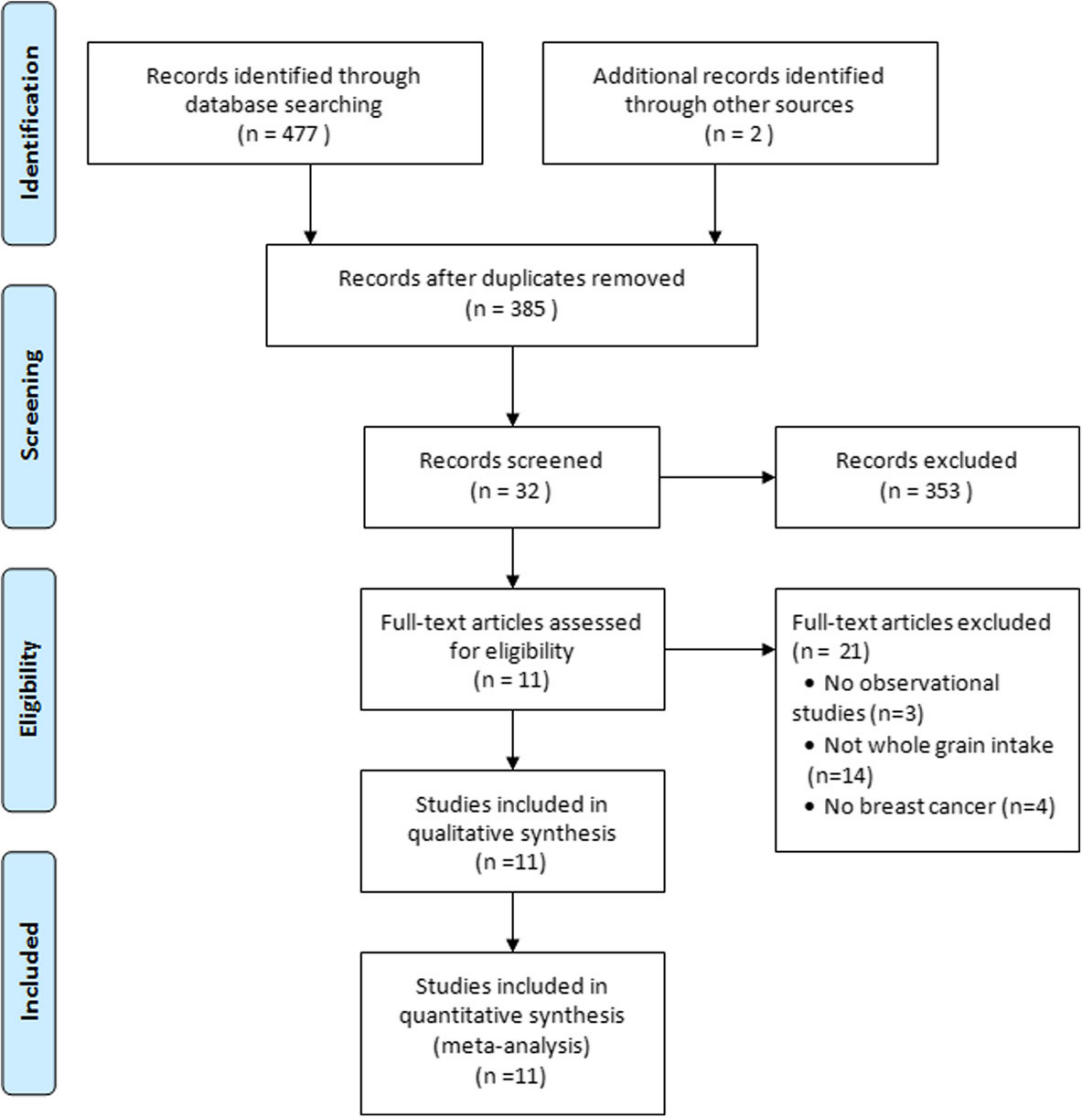
Flow chart of study selection. Flow chart shows literature search for whole grain intake in relation to risk of breast cancer

The search strategy identified 479 potentially relevant articles from the various databases, and 94 records were excluded because they were duplicates (Fig. 1). After title and abstract review based on the above inclusion criteria, 353 articles were further excluded. After reviewing the full texts of the remaining 32 articles, 21 papers were excluded, because 1) the studies were not case-control or cohort studies (*n* = 3); [10, 12, 28] 2) the studies did not assess the whole grain intake (*n* = 14); [29–42] or 3) the cases included were not breast cancer cases (*n* = 4) [12, 43–45]. Eleven studies involving 131,151 participants and 11,589 breast cancer cases were included in the present meta-analysis.

### Data extraction and quality assessment

Two reviewers independently extracted data on study characteristics and results by using a standard data collection form. Data extracted included: first author’s last name; year of publication; country of origin; study design; sample size; mean age of study population; dietary assessment methods; types of whole grain; RRs, including hazard ratios (HRs), ORs or incidence density ratios (IDRs), with the corresponding 95% CIs; and adjusted variables.

We also systematically assessed the study quality. Briefly, a 9-score system on the basis of the Newcastle Ottawa Scale (NOS) was used to assess the quality of included studies. Each study was evaluated on three broad criteria: 1) the proper selection of study population, 2) the comparability of the study groups, and 3) the ascertainment of the exposure or outcome of interest. Two reviewers independently assessed the quality of each study. Studies scored greater or equal to7 (out of a maximum 9 points) were considered to be high quality studies. Any discrepancies in data extraction and quality assessment between the reviewers were resolved by consensus.

### Statistical analyses

RR was used as a common measure of the association between whole grain intake and the risk of breast cancer. HRs, ORs, or IDRs were considered as estimates of RR. To calculate summary RR and its 95% CI, we pooled the results by using the random-effects meta-analysis [46]. The random-effects method was chosen a priori because of the anticipated clinical heterogeneity and because it is considered as more conservative than the fixed-effects method, as it accounts for both within- and between-study heterogeneity [47]. Heterogeneity across studies was evaluated by using the Q statistic with a conservative *p* value < 0.10 considered as statistically significant. We also calculated the *I*^2^ statistic, which describes the proportion of total variation across studies which was attributable to heterogeneity rather than chance alone; an *I*^2^ value greater than 50% indicated at least moderate heterogeneity [48]. Dose response relationship between whole grain intake and risk of breast cancer was analyzed by random-effects model and meta-regression with whole grain intake as an continuous variable. Furthermore, we assessed the influence of each individual study on the overall risk estimate by excluding one study at a time. Because characteristics of participants, and adjustments for confounding factors were not consistent across studies, we further conducted several sensitivity and stratified analyses to explore possible sources of heterogeneity and to examine the influence of various factors on the overall risk estimate. Subgroup analyses were performed by study design, sample size, publication year, numbers of adjusted variables, and quality scores of studies. Meta-regression analyses was used to evaluate the association of whole grain intake and risk of breast cancer between the subgroups.

Potential publication bias was evaluated by visual inspection of the Begg funnel plots in which the log RRs were plotted against their standard errors (SEs). We also performed the Begg rank correlation test and Egger linear regression test at the *p* < 0.10 level of significance [49, 50]. All analyses were performed using STATA version 11.0 (Stata Corp LP, College Station, Texas). *p* value < 0.05 was considered statistically significant, except those specified otherwise.

## Results

### Study characteristics

The 11 included studies were published between 1987 and 2016, among which four were cohort studies [15, 19, 22, 23] and seven were case-control studies [16–18, 20, 21, 24, 25] (Table 1). Two studies were conducted in the USA [15, 22], two in Italy [21, 24], and one in Greece [16], Iran [18], Denmark [23], German [20], Korea [17], Sweden [19], and Switzerland [25], respectively. The age of the participants ranged from 25 to 75. The studies were adjusted for a wide range of potential confounding factors, including age, BMI, menopausal status, family history of breast cancer, hormone use, physical activity, smoking, energy intake, etc. The type and dose of whole grain intake and the relative risk of breast cancer are presented in Table 2.

**Table 1:** Descriptions of the studies included in the systematic review and meta-analysis of whole grain intake and breast cancer risk

Study	Location	Design	Sample size	Age	Diet-assessment method	Adjustment variables
Farvid et al., 2016 [15]	United States	NHSII Cohort (22)	44,263(3235 cases)	36 ± 5	Adult diet was evaluated using FFQ (130 items, past year); Adolescent diet was evaluated using 124-item high school FFQ (1960–1980)	Age, smoking, race, parity and age at first birth, height, BMI, weight, family history of breast cancer, history of benign breast disease, oral contraceptive use, adult alcohol intake, physical activity, energy intake, hormone use and menopausal status, age at menopause.
Mourouti et al., 2016 [16]	Greece	Case-control	250 cases/250 controls	56 ± 12	FFQ (86 items, last year prior to diagnosis)	Age, BMI, International Physical Activity Questionnaire, Smoking ever, Menopausal status, Family history of breast cancer, MedDietScore.
Tajaddini et al., 2015 [18]	Iran	Case-control	306 cases/309 controls	25–65	FFQ(136 items, a previous year before diagnosis for cases or before interview for controls)	Age at diagnosis, menopause, total calorie, parity, and BMI.
Yun et al., 2010 [17]	Korea	Case-control	362 cases/362 controls	30–65	quantitative food frequency questionnaire (FFQ) with 121 items	BMI, alcohol drinking, multivitamin use, number of children, breast feeding, and dietary factors including soy protein, folate, vitamin E, and fiber.
Egeberg et al., 2009 [23]	Denmark	Danish Diet, Cancer and Health cohort study (9.6)	25,278 (978 cases)	50–64	FFQ(192 items, at baseline 1993–1997)	Parity (parous/nulliparous and number of births), age at first birth, education, duration of hormone replacement therapy use, use of hormone replacement therapy, intake of alcohol and BMI.
Sonestedt et al.,2008 [19]	Sweden	Malmo Diet and Cancer cohort(10.3)	15,773(544 cases)	46–75	a 168-items dietary questionnaire	Season of data collection, diet interviewer, method version, age, total energy, weight, height, educational status, smoking habits, leisure time physical activity, hours of household activities, alcohol consumption, age at menopause, parity and current use of HRT.
Adzersen et al., 2003 [20]	Germany	Case-control	310 cases/353 controls	25–75	FFQ (161items, Hospital interview)	Age, total energy without alcohol intake, age at menarche, age at first birth, age at menopause, mother/sister with breast cancer, current smoking, history of benign breast disease and/or operation, BMI, consumption of alcohol, current HRT or HRT during the past year.
Nicodemus et al.., 2001 [22]	United States	Cohort Iowa Women’s Health Study(9)	29,119 (977 cases)	55–69	a standard FFQ and an additional question that asked for the type of breakfast cereal usually eaten	Age, energy intake, estrogen use, personal history of benign breast disease, family history of breast cancer, mammography status, age at first live birth, number of live births, current weight, waist-to-hip ratio, vitamin use, educational attainment, vitamin A and refined grain intake.
Chatenoud et al., 1998 [21]	Italy	Case-control	3412 cases/7990 controls	< 74	FFQ(14-37items, during the 2 years before diagnosis for cases or before interview for controls)	Age, sex, education, smoking habits, alcohol intake and BMI.
Levi et al., 1993 [25]	Switzerland	Case-control	107 cases/318 controls	30–75	Hospital interview, FFQ (50 foods, since 1990)	Age, sex, education, BMI, physical activity, energy, parity.
LaVecchia et al., 1987 [24]	Italy	Case-control	1108 cases/1281 controls	25–74	Frequency of consumption of major food sources year before interview of first symptoms (1979–1984)	Age, sex, education, green vegetables, fresh fruit, 7 reproductive variables, history of benign breast cancer for patient, mother, and sisters.

*FFQ* food frequency questionnaire, *BMI* body mass index, *HRT* hormone replacement therapy

**Table 2:** The type and dose of whole grain intake and the relative risk of breast cancer in the included studies

Study	Type of whole grain intake	Dose of whole grain intake (g/d)	Relative Risk(95%CI)
Farvid et al., 2016 [15]	whole grain foods	Q1: 5.6; Q2: 14; Q3: 19.6; Q4: 28; Q5: 42	Q1: 1; Q2: 0.93 (0.83–1.03); Q3: 0.87 (0.77–0.97); Q4: 0.91 (0.81–1.02); Q5: 0.91 (0.81–1.03)
Mourouti et al., 2016 [16]	whole grain foods (including whole grain bread, whole grain cereals, oatmeal, whole wheat pasta, brown or wild rice)	No reported	Never/rarely: 1; 1–6 times/week: 0.68 (0.41, 1.09); > 7 times/week:0.49 (0.29, 0.82)
Tajaddini et al., 2015 [18]	whole-wheat bread (Sangak, Taftoon, Barbari, barley, corn flakes and sprouts)	< 1.0; 1.0–23.0; > 23.0	< 1.0 g/d: 1; 1.0–23.0 g/d: 1.39(0.68–2.83); > 23.0 g/d: 0.61(0.37–0.99)
Yun et al., 2010 [17]	mixed brown rice	0; 100; 350	0 g/d:1.0; 100 g/d: 0.90(0.47,1.71); 350 g/d: 0.42(0.20,0.87) Per 100 g/d: 0.76(0.61,0.95)
Egeberg et al., 2009 [23]	whole grain products (rye bread, whole grain bread and oatmeal)	≤72; 72 to ≤112; 112 to ≤163; > 163	≤72 g/d: 1; 72 to ≤112 g/d: 0.98 (0.82–1.17); 112 to ≤163 g/d: 1.00 (0.85–1.19); > 163 g/d: 1.03 (0.85–1.24) Per each additional 50 g/day: 1.01(0.96–1.07)
Sonestedt et al.,2008 [19]	high-fibre bread (≥ 6% of fibre for soft bread, ≥10% for crisp bread and ≥ 10% for biscuits and rusks)	Q1: 0; Q2:9; Q3:19; Q4: 34; Q5: 65	Q1: 1; Q2: 0.87 (0.67–1.13); Q3: 0.74 (0.56–0.97); Q4: 0.82 (0.63–1.07); Q5: 0.75 (0.57–0.98)
Adzersen et al., 2003 [20]	the whole-grain category all whole-grain bread and rice, rolled oats, muesli, and cornflakes.	Q1: < 18.3; Q2:18.3 ≤ 32.6; Q3: 32.6 ≤ 45.5;Q4:> 45.5	Q1: 1; Q2: 0.96 (0.61,1.52); Q3:0.76 (0.47–1.24); Q4:0.57 (0.34–0.95)
Nicodemus et al., 2001 [22]	whole grains	Q1:0–3.5; Q2: 4–7; Q3: 7.5–10.5; Q4:11–18.5; Q5:19–108.5 (servings/week)	Q1: 1; Q2: 0.95 (0.76–1.2); Q3: 1.04 (0.84–1.3); Q4: 1.19 (0.96–1.5); Q5: 1.21 (0.96–1.5)
Chatenoud et al., 1998 [21]	whole grain food (essentially bread or pasta)	No reported	Low (no or rare consumption): 1; Intermediate (1–3 days/week): 0.9(0.8–1.0);High (> 3 days/week): 0.9(0.8–1.0)
Levi et al., 1993 [25]	whole-grain bread and pasta	No reported	Low: 1; Intermediate: 0.77(0.41–1.44); High: 0.63(0.35–1.15)
LaVecchia et al., 1987 [24]	whole-grain bread or pasta	No reported	Never: 1; Occasionally: 0.75(0.57–0.96); Frequently: 0.90(0.69–1.17)

Q = quintiles or quartiles

According to the NOS criteria, the quality scores of the included studies ranged from 6 to 9 (Tables 3 and 4). Nine studies were considered as high-quality and two as low-quality studies. Most case-control studies had exposure assessment and selection biases and did not report the non-response rates.

**Table 3:** Assessment of study quality included in the meta-analysis by Newcastle Ottawa Scale (NOS) for case-control studies

Source	Selection				Comparability^a^		Exposure			Total scores
	1	2	3	4	5A	5B	6	7	8	
Mourouti et al.	*	*	*	*	*	*	*	*	–	8
Tajaddini et al.	*	*	–	*	*	*	*	*	–	7
Yun et al	*	*	–	–	*	*	*	*	–	6
Adzersen et al.	*	*	–	*	*	*	–	*	–	6
Chatenoud et al.	*	*	–	*	*	*	–	*	*	7
Levi et al.	*	–	–	*	*	*	*	*	*	7
LaVecchia et al	*	*	–	*	*	*	*	*	*	8

1 Is the case definition adequate? 2 Representativeness of the cases. 3 Selection of controls. 4 Definition of controls. 5 Comparability of cases and controls on the basis of the design or analysis. 6 Ascertainment of exposure. 7 Same method of ascertainment for cases and controls. 8 Non-response rate

^a^Studies that controlled for age and traditional risk factors received one score, whereas studies that controlled for other important confounders received an additional score

**Table 4:** Assessment of study quality included in the meta-analysis by Newcastle Ottawa Scale (NOS) for cohort studies

Source	Selection				Comparability^a^		Outcome			Total scores
	1	2	3	4	5A	5B	6	7 ^b^	8 ^c^	
Farvid et al	–	*	*	*	*	*	*	*	*	8
Egeberg et al.	*	*	–	*	*	*	*	*	–	7
Sonestedt et al	*	*	*	*	*	*	*	*	*	9
Nicodemus et al.	*	*	*	*	*	*	*	*	–	8

1 Representativeness of the exposed cohort. 2 Selection of the non-exposed cohort. 3 Ascertainment of exposure for cohort studies. 4 Demonstration that outcome of interest was not present at start of study for cohort studies. 5 Comparability of cohorts on the basis of the design or analysis. 6 Assessment of outcome. 7 Was follow-up long enough for outcomes to occur. 8 Adequacy of follow up of cohorts

^a^Studies that controlled for age and traditional risk factors received one score, whereas studies that controlled for other important confounders received an additional score

^b^study with follow-up time > 2 years was assigned one score

^c^study with follow-up rate > 70% was assigned one score

### Associations between whole grain intake and the risk of breast cancer

**Fig. 2:**
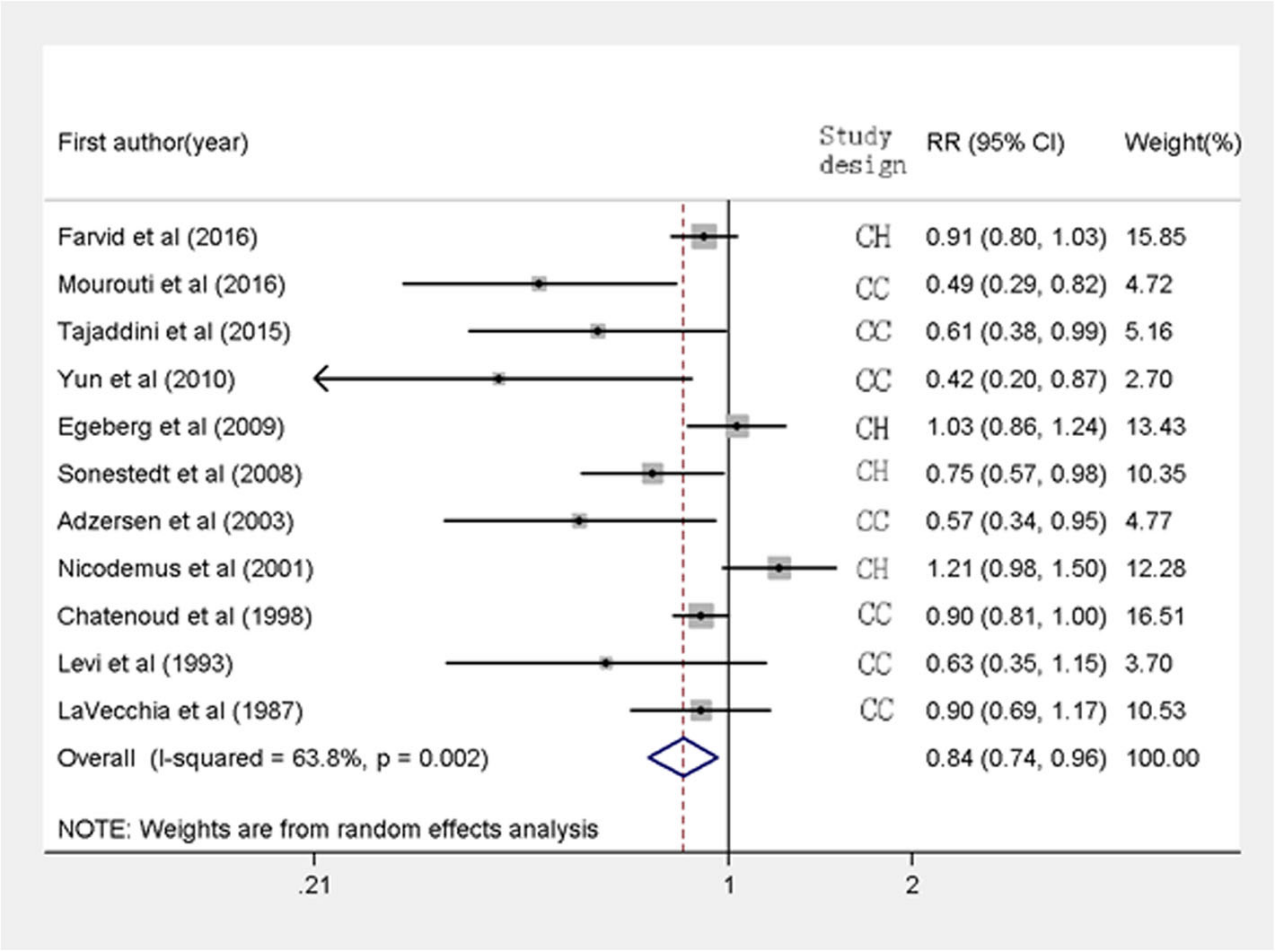
Forest plot shows the association between highest category of whole grain intake and the risk of breast cancer. CH, cohort study, CC, case-control study

The RRs of breast cancer risk comparing the highest versus the lowest levels of whole grain intake varied from 0.42 to 1.21 across the studies (Fig. 2). Five studies showed a significant inverse association [16–20], one study reported a marginally significant association [21], whereas no association was found in the remaining five studies [15, 22–25]. When the results were combined by using random-effects method, a significant inverse association was observed (RR: 0.84, 95% CI: 0.74 to 0.96, *p* = 0.009), with significant heterogeneity (*I*^2^ = 63.8%, *p* = 0.002).

**Fig. 3:**
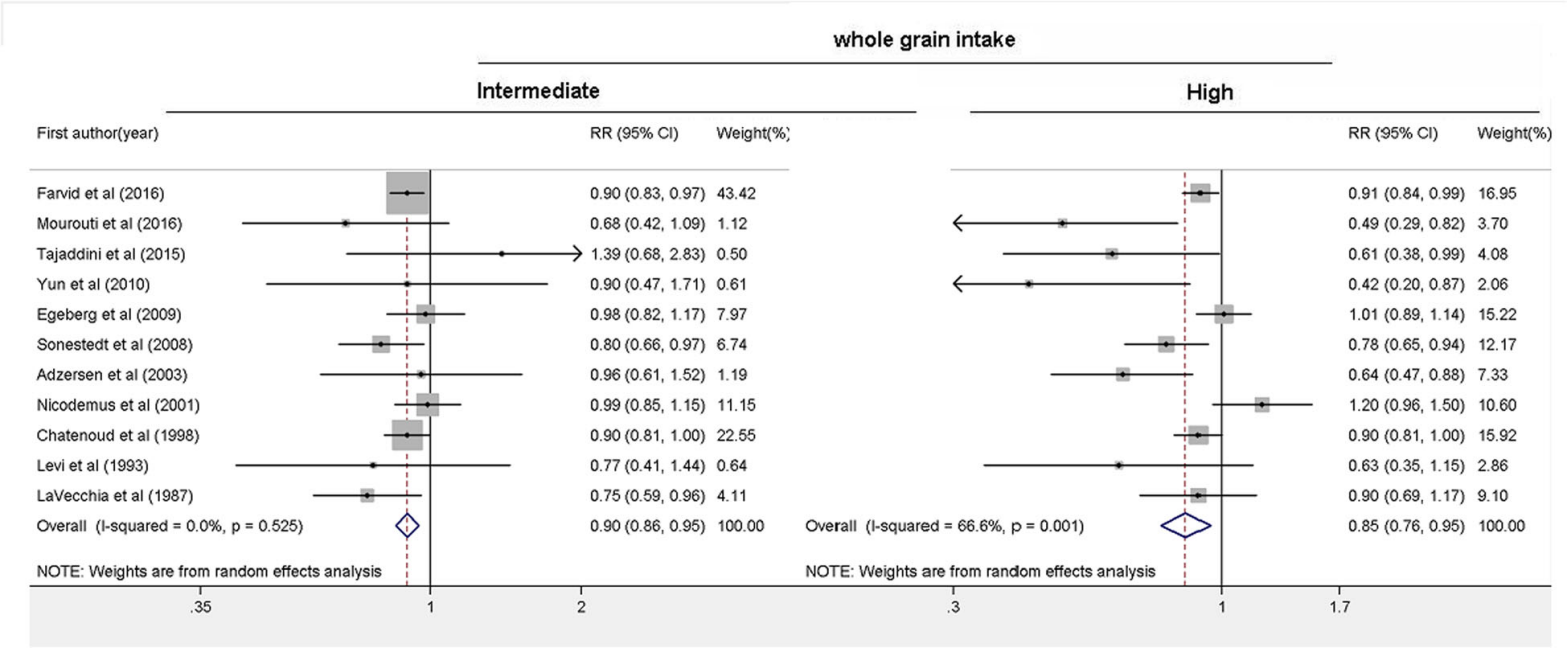
Forest plot shows the association between high and intermediate levels of whole grain intake and the risk of breast cancer

**Fig. 4:**
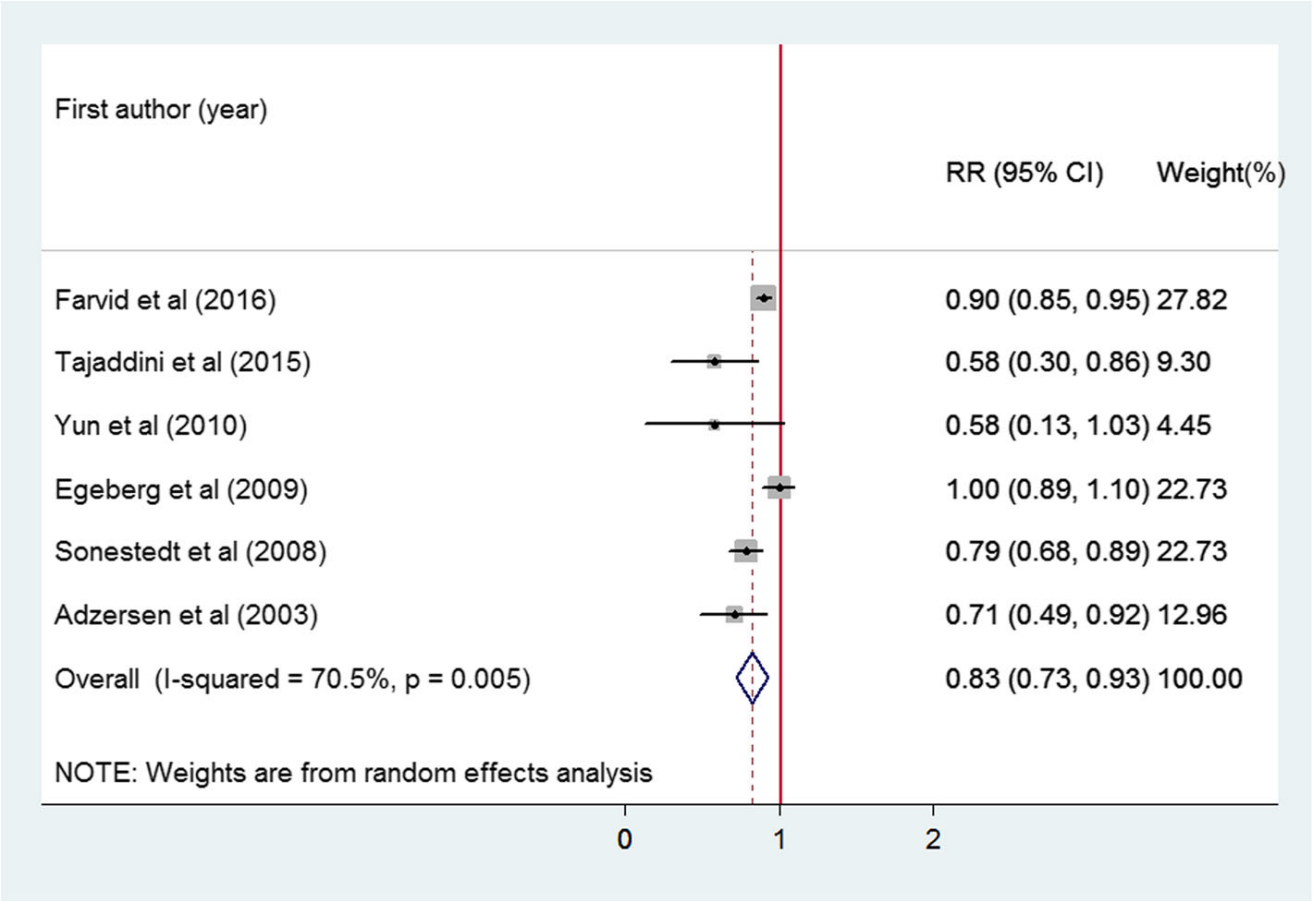
Forest plot shows the association between whole grain intake (per 50 g/day) as a continuous variable and the risk of breast cancer

**Fig. 5:**
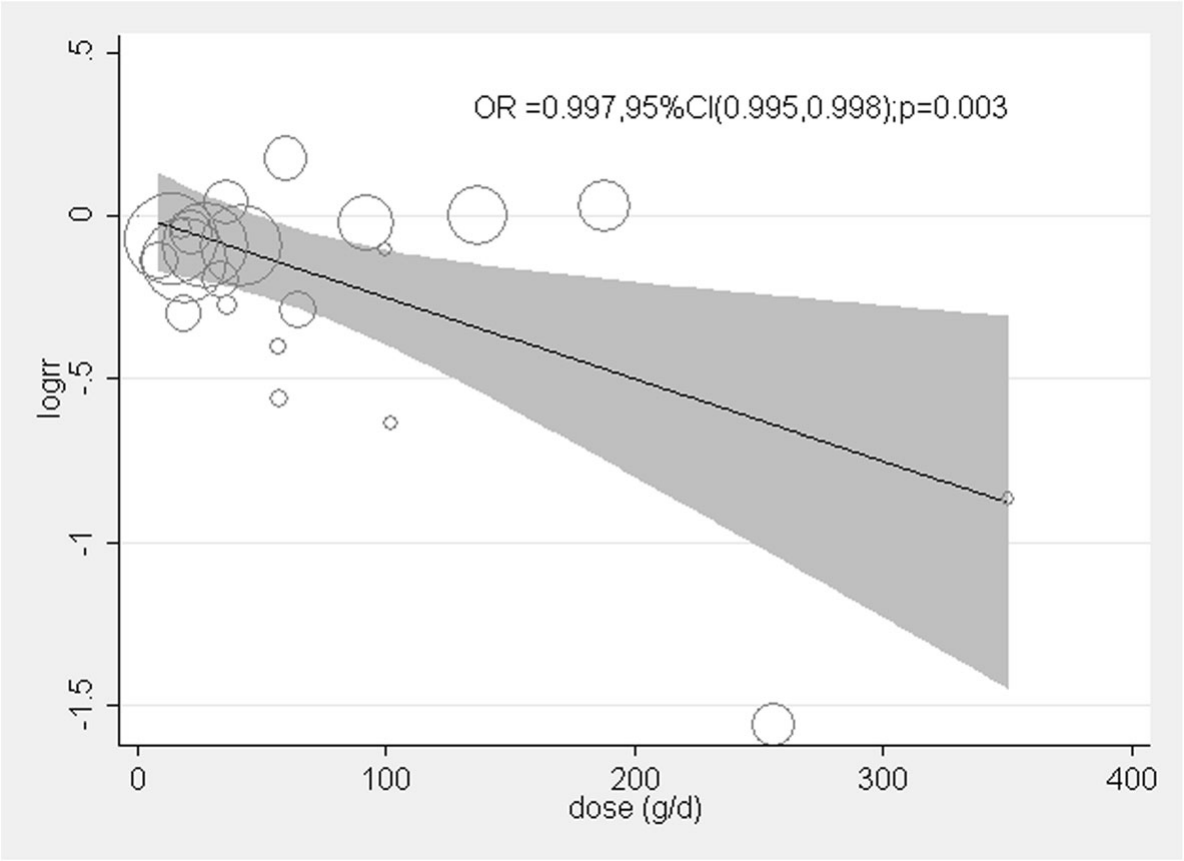
Meta regression analysis of the association between the dose of whole grain intake and the risk of breast cancer

Whole grain intake was classified in different ways in the included studies, quintiles in three studies [15, 19, 22], quartiles in two studies [20, 23], and tertiles in the other six studies. We combined quintile 2 and 3 into intermediate intake level, quintile 4 and 5 and quartile 3 and 4 into high level, to further quantify the associations of different intake levels of whole grain intake with breast cancer risk. A significant inverse association was found for both the intermediate intake level (RR: 0.90, 95%CI: 0.86, 0.95; *p* < 0.001) and the high intake level of whole grains (RR: 0.85, 95% CI: 0.76, 0.95, *p* = 0.004). No significant heterogeneity was observed for the intermediate intake level (*I*^2^ = 0.0%, *p* = 0.525), whereas significant heterogeneity was found for the high intake level (*I*^2^ = 66.6%, *p* = 0.001) (Fig. 3). Six studies reported the intake of whole grain as a continuous variable (g/d). The pooled analysis showed that an average 50 g/d intake of whole grain was significantly associated with a 17% reduced risk of breast cancer with significant heterogeneity (RR: 0.83, 95% CI: 0.73, 0.93; *I*^2^ = 70.5%, *p* = 0.005) (Fig. 4). To explore the association between the dose of whole grain intake and breast cancer risk, we further performed a meta-regression analysis and found an inverse association between the dose of whole grain and breast cancer risk (Fig. 5).

### Stratified and sensitivity analyses

Stratified analysis by study design found a significant inverse association between whole grain intake and breast cancer risk in the seven case-control studies (RR:0.69, 95% CI: 0.56 to 0.87, *p* = 0.001; *I*^2^ = 58.2%, *p*_for heterogeneity_ = 0.026), but no association in the four cohort studies (RR: 0.96, 95% CI: 0.82 to 1.14, *p* = 0.69; *I*^2^ = 66.7%, p _for heterogeneity_ = 0.029).In addition, a significant association was observed in studies with sample size ≤2300 (RR: 0.55, 95% CI: 0.43 to 0.70, *p* < 0.001), those with the number of adjusted variables ≤7 (RR: 0.80, 95% CI: 0.64 to 0.99, *p* = 0.04), studies published before 2008 (RR: 0.75, 95% CI: 0.58 to 0.97, *p* = 0.03), or studies with quality score ≤ 7 (RR: 0.77, 95% CI: 0.63 to 0.96, *p* = 0.019). To explore whether these associations were statistically different between the subgroups, we further performed meta-regression analyses and found the association was statistically different between the subgroups of sample size (*p* < 0.05) but was not significant between other subgroups (Additional file 2: Figure S1). Significant heterogeneity was observed in different subgroups based on publication year, numbers of adjusted variables, and quality score. However, no significant heterogeneity was observed in subgroups based on sample size, suggesting sample size may be a possible source of heterogeneity across the studies (Table 5).

**Table 5:** Subgroup analyses for the association between whole grain intake and breast cancer risk

Subgroups	No. of studies	RR (95% CI)	*P*	Heterogeneity χ^2^	*I*^2^(%)	*P for heterogeneity*
Overall	11	0.84 (0.74,0.96)	0.009	27.6	63.8	0.002
Study design						
Cohort study	4	0.96 (0.82,1.14)	0.69	9.0	66.7	0.029
Case-control study	7	0.69 (0.56, 0.87)	0.001	14.3	58.2	0.026
Sample size						
≤ 2300, below median	5	0.55 (0.43, 0.70)	< 0.001	1.1	0.0	0.893
> 2300, above median	6	0.94 (0.85,1.04)	0.25	9.9	49.8	0.077
Publication year						
After 2008	6	0.87 (0.74,1.03)	0.12	13.4	62.9	0.019
Before 2008	5	0.75 (0.58,0.97)	0.032	14.1	71.6	0.007
Number of adjustment for covariates						
≤ 7	5	0.80 (0.64,0.99)	0.04	11.1	64.2	0.025
> 7	6	0.84 (0.69,1.04)	0.11	16.3	69.4	0.006
Study quality score						
≤ 7	6	0.77 (0.63, 0.96)	0.019	13.0	61.6	0.023
> 7	5	0.87 (0.71,1.07)	0.19	14.3	72.0	0.006

Sensitivity analyses were also conducted to assess the influence of each individual study on the summary estimates by eliminating one study at a time. The results suggest that the estimates were robust, with the summary RRs ranging from 0.86 to 0.90 and all *p* values < 0.05 (Additional file 3: Figure S2).

### Publication bias

Although the funnel plot was slightly asymmetric, after using the trim-and-fill method, visual inspection of the Begg funnel plot did not identify substantial asymmetry (Additional file 4: Figure S3). In addition, the Begg rank correlation test and Egger linear regression test showed no evidence of publication bias (Begg test, *p* = 0.300; Egger test, *p* = 0.309).

## Discussion

To the best of our knowledge, this is the first meta-analysis of observational studies to quantitatively summarize the evidence of the association between whole grain intake and the risk of breast cancer. The results suggest that intermediate and high intake levels of whole grain were associated with a modest reduction of breast cancer risk. The meta-regression analysis found an inverse association between the dose of whole grain intake and the risk of breast cancer. In addition, stratified analyses found this inverse association was significant in case-control studies, but not in cohort studies.

In 1987, La Vecchia et al. [24] first reported that the intake of whole grain bread was inversely associated with the risk of breast cancer in a case-control study conducted in Italy. Subsequently, another case-control study published in 1993 by Levi et al. [25] did not find a significant association. In 1998, Jacobs et al. [14] conducted a meta-analysis of 40 case-controls studies including 20 cancer sites and found that whole grain consumption was protective against different types of cancer, such as colon cancer, gastric cancer, and pancreatic cancer. Because only the above two case-control studies were included in that review and meta-analysis, no significant association was observed for breast cancer. Since then, nine observational studies have published with inconsistent results reported. In the present meta-analysis including 11 observational studies, we found that whole grain intake was significantly inversely associated with breast cancer risk.

Several mechanisms have been proposed to explain the reduced risk of breast cancer with whole grain intake. Whole grains contain various micronutrients and are rich in non-nutrients that are lost in the refining process but may be potentially beneficial in preventing cancer [51, 52]. First, whole grains may reduce the postprandial glucose and insulin responses leading to better glycemic control [53]. Higher serum insulin levels have been found to be associated with an increased breast cancer risk in several epidemiological studies [54, 55]. Therefore, insulin and glycemic control could be a potential pathway through which whole grains may reduce breast cancer risk. Whole grain has also been found to be associated with reduced levels of inflammatory markers (plasminogen activator inhibitor-1, C-reactive protein) and liver enzymes (gamm-glutamyltranspeptidse, aspartate aminotransferase) [56], and higher levels of these markers and enzymes were associated with an increased risk of cancer [57]. Second, whole grains are a rich source of dietary fiber. A recent meta-analysis of 16 prospective studies found that dietary fiber intake was inversely associated with breast cancer risk [13]. High fiber foods are known to have potential anticarcinogenic properties, for instance, reducing N-nitroso compounds, enhancing immunity, and particularly producing various anti-inflammatory cytokines, which may be involved in the initiation and progression of breast cancer [58]. Dietary fibre can reduce cancer risk through removing damaged cells from the digestive tract [59], increasing stool bulk, diluting carcinogens, decreasing transit time, altering the gut microbiota [60–62], and binding oestrogens in the colon and increasing the faecal excretion of oestrogens, leading to lower oestrogen concentrations [63]. In addition, dietary fiber can bind to or dilute bile acids to reduce cell proliferation and the chance of mutations [64]. Third, whole grains are rich in antioxidants, including vitamins (vitamin C and E and β-carotene) and trace minerals (selenium, zinc, copper, and manganese), which are components of enzymes with antioxidant functions. Vitamin E inhibits cancer through preventing carcinogen formation and blocking carcinogen-cell interactions [65]. These vitamins and trace minerals have been found to be inversely associated with breast cancer risk [56]. Finally, whole grains are a significant source of some essential non-nutrients, such as phytoestrogens, phenolic acids, and lignans. These natural compounds play important protective roles against cancer through their antioxidant properties and abilities to inhibit cell proliferation and angiogenesis and to induce cell apoptosis, as well as through modulating hormonal pathways [66]. Although these mechanisms are biologically plausible, it is difficult to determine the specific bioactive components of whole grains which contribute to breast cancer risk reduction in epidemiologic research. Further experimental studies are needed to confirm the underlying mechanisms through which whole grains or the bioactive components reduce breast cancer risk.

Heterogeneity poses an important challenge in conducting and interpreting the results of meta-analyses [67]. Various factors may contribute to heterogeneity. As the overall results of our meta-analysis revealed significant heterogeneity across the included studies, subgroup analyses were conducted to find the potential sources. The results showed significant heterogeneity in subgroups stratified by study design, publication year, the number of adjusted covariates, and study quality score (all *p* < 0.05). However, the stratified analyses by sample size found that heterogeneity was no longer significant in the two subgroups (*I*^2^ = 0% and 49.8%, both *p* > 0.05), suggesting that sample size might be a potential source of heterogeneity. In addition, the association was only significant in case-control studies, in studies with sample size≤2300, published before 2008, or studies with the number of adjusted covariates ≤7 or quality score ≤ 7, but not significant in cohort studies, in studies with sample size> 2300, published after 2008, or studies with the number of adjusted covariates > 7 or with quality score > 7. Approximately two thirds of the studies were case-control studies with inadequate adjustment of potential confounders and comparatively low quality. Given that the possible recall bias and selection bias in case-control studies, and the limited number of only four cohort studies, more large-scale prospective cohort studies with full adjustment for potential confounding factors are urgently needed to confirm the inverse association observed in the current meta-analysis.

Our meta-analysis has several limitations. First, it included seven case-control studies and four cohort studies. As indicated above, case-control studies are likely susceptible to recall and selection bias. Second, the quality of the included studies was moderate and the inverse association was only observed in low quality studies (NOS ≤ 7, *n* = 6) in the subgroup analysis by quality score. Third, the intake levels of whole grain were reported in six included studies. However, a dose-response meta-analysis could not be conducted due to incomplete data and the inconsistencies in the measurement units of whole grain intake and different assessment methods. We did perform a meta-regression analysis to explore the association between dose of whole grain intake and breast cancer risk and found an inverse association. In addition, differences in the definitions of whole grain and in the categories of whole grain foods among studies might also be another possible source of heterogeneity. Fourth, the 95%CI was not reported in one study [25] and was extracted from a previous meta-analysis [14] which may result in inaccurate estimates. Finally, although most included studies adjusted for major potential confounders, other unmeasured and uncontrolled confounders, such as coffee [68] and green tea consumption [69], may potentially affect the validity of the results to some extent.

## Conclusions

Dietary intake of whole grains was inversely associated with breast cancer risk in the current meta-analysis, and the inverse association was only observed in case-control but not cohort studies. Considering a limited number of case-control studies, the potential biases of case-control studies, and that sample size may be a potential source of heterogeneity, large well-designed prospective cohort studies need to be conducted. Future studies should further elucidate the dose-response relationship and assess the associations of whole grain and whole wheat with breast cancer.

## Additional files

Additional file 1: PRISMA 2009 Checklist. (DOC 64 kb)

Additional file 2: **Figure S1.** Meta regression analysis of the association between the publication year (A, *p* = 0.43), the sample size (B, *p* = 0.04), the number of adjustment covariates (C, *p* = 0.36), and the study quality score (D, *p* = 0.32) and the risk of breast cancer. (TIF 62 kb)

Additional file 3: **Figure S2.** Sensitivity analyses of the association between whole grain intake and the risk of breast cancer with one study omitted at a time. (TIF 7 kb)

Additional file 4: **Figure S3.** Publication bias assessed by funnel plot of the association between whole grain intake and the risk of breast cancer in the meta-analysis. (TIF 10 kb)

## Abbreviations

CI: Confidence interval
HRs: Hazard ratios
IDRs: Incidence density ratios
MESH: Medical subject headings
NOS: Newcastle Ottawa Scale
ORs: Odds ratios
RR: Relative risk
SEs: Standard errors
