# Supplementary figures and images for: Association between whole grain intake and breast cancer risk: a systematic review and meta-analysis of observational studies
Source: Nutr J. 2018 Sep 21;17:87. doi: 10.1186/s12937-018-0394-2 (PMC6201708; doi:10.1186/s12937-018-0394-2)

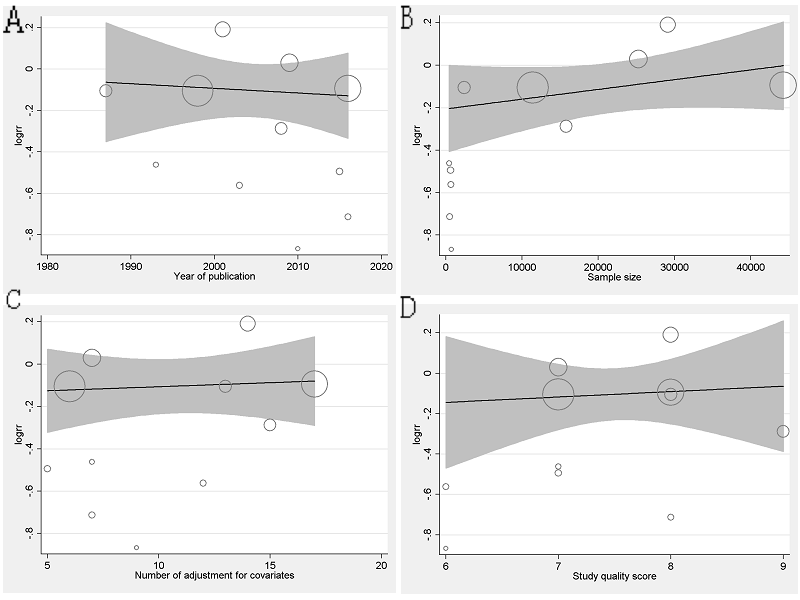

Supplement: Supplementary file 2 — Figure S1. Meta regression analysis of the association between the publication year (A, p = 0.43), the sample size (B, p = 0.04), the number of adjustment covariates (C, p = 0.36), and the study quality score (D, p = 0.32) and the risk of breast cancer. (TIF 62 kb) [file 12937_2018_394_MOESM2_ESM.tif]
